# Porphyrins inactivate *Nosema* spp. microsporidia

**DOI:** 10.1038/s41598-018-23678-8

**Published:** 2018-04-03

**Authors:** Aneta A. Ptaszyńska, Mariusz Trytek, Grzegorz Borsuk, Katarzyna Buczek, Katarzyna Rybicka-Jasińska, Dorota Gryko

**Affiliations:** 1 Department of Botany and Mycology, Institute of Biology and Biochemistry, Faculty of Biology and Biotechnology, Maria Curie-Skłodowska University, Akademicka 19, 20-033 Lublin, Poland; 2 Department of Industrial Microbiology, Institute of Microbiology and Biotechnology, Faculty of Biology and Biotechnology, Maria Curie-Skłodowska University, Akademicka 19, 20-033 Lublin, Poland; 3 Institute of Biological Basis of Animal Production, Faculty of Biology, Animal Sciences and Bioeconomy, University of Life Sciences in Lublin, Akademicka 13, 20-950 Lublin, Poland; 4 Institute of Organic Chemistry, Polish Academy of Sciences, Kasprzaka 44/52, 01-224 Warsaw, Poland

## Abstract

The study of organic/inorganic molecules with activity against intracellular fungi of the phylum Microsporidia is of critical importance. Here, for the first time, the inactivation of these parasitic fungi by porphyrins is reported. The biological effects of porphyrins (10 µM and 100 µM) on the microsporidian *Nosema ceranae* was investigated in honeybee hosts using cage experiments. A significant reduction in the number of spores (from 2.6 to 5 fold) was observed in *Nosema*-infected honeybees with a sucrose-protoporphyrin amide [PP(Asp)_2_] syrup diet compared to the control honeybees. PP(Asp)_2_ and the other porphyrin examined *in vitro*, TMePyP, had a direct impact on the microsporidia. Notably, neither porphyrin requires light excitation to be active against microsporidia. Moreover, microsporidia preincubated with these porphyrins exhibited decreased ability to infect honeybees. In particular, PP(Asp)_2_, possessing amphiphilic characteristics, exhibited significant inactivation of microsporidia, preventing the development of the microsporidia and diminishing the mortality of infected honeybees. In addition, the porphyrin-treated spores examined by scanning electron microscopy (SEM) showed morphological changes in their exosporium layers, which were distinctly deformed. Thus, we postulate that the mechanism of action of porphyrins on microsporidia is not based on photodynamic inactivation but on the destruction of the cell walls of the spores.

## Introduction

Pathogenic fungi from the phylum Microsporidia are intracellular parasites found in both vertebrates and invertebrates, including honeybees. Some species are responsible for a number of human diseases that are predominantly associated with immune suppression^1^. *Enterocytozoon bieneusi* is recognized as one of the most opportunistic human pathogens, causing microsporidiosis, which results in potentially fatal diarrhoea and “wasting syndrome”^2,3^.

*Nosema apis* and *Nosema ceranae* are particularly devastating to honeybees as they complete their life cycle in the intestinal tracts of honeybees, leading to anatomical and physiological changes that result in the deterioration of honeybee health and, ultimately, to the total collapse of a colony^4–6^.

The only effective medicinal treatment for the microsporidia *Nosema and Enterocytozoon* is the antibiotic fumagillin^7,8^. Fumagillin and its derivatives exhibit activity against several groups of parasites; however, recent studies have demonstrated the severe toxic side effects of fumagillin (neutropenia and thrombocytopenia) in human subjects^9,10^. Furthermore, *N. ceranae* has been found to become resistant to fumagillin over time^11^. Other known inactivators of microsporidiosis include benzimidazoles such as albendazole and polyamine analogues^2,8^. Despite some positive reports, *in vitro* tests of these compounds against microsporidia that infect humans have been rather limited, with only a few species shown to be sensitive to these agents. Therefore, investigations into new, diverse and robust medicinal treatments to combat microsporidiosis are of critical importance. Our preliminary studies show a breakthrough in microsporidiosis treatment, for which porphyrinoids were found to be very promising candidates.

Porphyrins, which are aromatic heterocyclic compounds, are ubiquitous in nature. These compounds participate in various biochemical processes in living organisms, such as oxygen transport (haem) and photosynthesis (chlorophylls). Non-metallated porphyrins and their metallated complexes have been used as therapeutic agents for many years, and the popularity of these compounds is rapidly increasing^12,13^. Porphyrins are promising alternatives to conventional antibiotics and have already been shown to be effective *in vitro* in the photodynamic inactivation of bacteria, viruses, fungi and protozoa^14,15^. The mode of action of this inactivation is based on the formation of reactive oxygen species (ROS) by light irradiation of a porphyrin in an environment containing molecular oxygen^16,17^.

Generally, non-metallated porphyrins are much more effective against microorganisms when irradiated than in the dark. Little is known about porphyrinoid-based inactivators of microorganisms (especially porphyrin sensitizers), specifically those operating in the absence of light excitation. For instance, gallium protoporphyrin inhibits the growth of a range of bacterial species by entering bacterial cells via a high-affinity haem transport system^18^. Recently, metalloporphyrins and metal-free porphyrins were found to exhibit rapid bactericidal activity against a range of clinically important *Staphylococcus aureus* isolates in the dark^19,20^ and were shown to act on both dividing and nongrowing bacteria within biofilms^21^. This finding clearly demonstrates the potential of porphyrins, primarily with respect to the emergence of mutational resistance at sub-inhibitory concentrations^20^.

Most photoactive compounds against *Saccharomyces cerevisiae* are known to be amphiphilic and are able to permeate cell walls and membranes, thus accessing internal targets^22^. The amide-linked deuteroporphyrin-nitroimidazole (DPIX-Nim) adduct has been shown to be very effective against the periodontal pathogen *Porphyromonas gingivalis*^23^. Hence, the conjugation of porphyrin molecules with amino acid moieties increases the potency, water solubility and uptake of these molecules compared to commercially available porphyrins, e.g., TMePyP. On the other hand, photoinactivation of fungal cells is mostly achieved using porphyrins that are present in the incubation medium rather than attached to the cells^14,23^.

To date, systematic studies on porphyrinoids with antifungal activity in the dark have yet to be conducted. Furthermore, to the best of our knowledge, there are no reported cases of microsporidiosis treatment with these compounds. Therefore, the aim of this study is to determine whether porphyrins are effective against pathogenic fungi from the phylum Microsporidia, i.e., *Nosema* spp., and to determine whether photosensitization plays a key role in the activity of these porphyrins.

The consequence of inhibition of *N. ceranae* infection by the synthetic amphiphilic protoporphyrin amide PP(Asp)_2_ (protoporphyrin conjugated to aspartate moieties) was studied in honeybee hosts. Porphyrins similarly characterized by increased bioavailability, i.e., amide-linked porphyrins and/or arylglycosylporphyrins, have been effective against some pathogens, but these porphyrins required the presence of nitroimidazole antibiotics or light, respectively^22,23^. Furthermore, to gain insight into the probable mechanism of action, the commercially available porphyrin TMePyP, with low capacity to penetrate microbial cell walls and membranes, was studied. We also investigated the direct effect of these compounds on the morphology of microsporidial cell walls and on the infectivity of pre-treated microsporidia.

## Results

**Figure 1:**
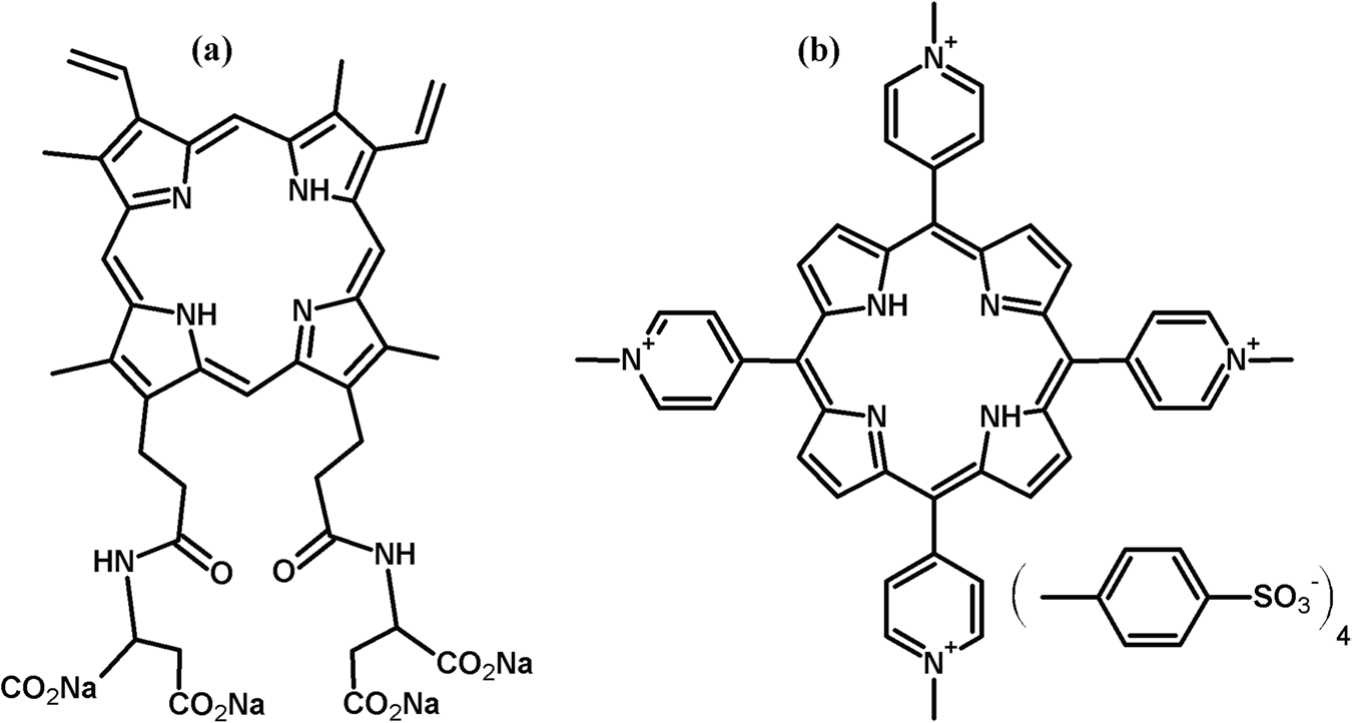
Molecular structures of the protoporphyrin IX derivative PP(Asp)_2_ (**a**) and TMePyP (**b**).

The amphiphilic protoporphyrin amide derivative PP(Asp)_2_, possessing aspartic amide moieties (Fig. 1), was chosen for the initial experimental studies. The effect of this porphyrin on microsporidiosis development and on the survival of both healthy and *Nosema*-infected honeybees was examined (Experiment 1). This experiment was designed to gain insight into the toxic effects of PP(Asp)_2_ on honeybees.

**Figure 2:**
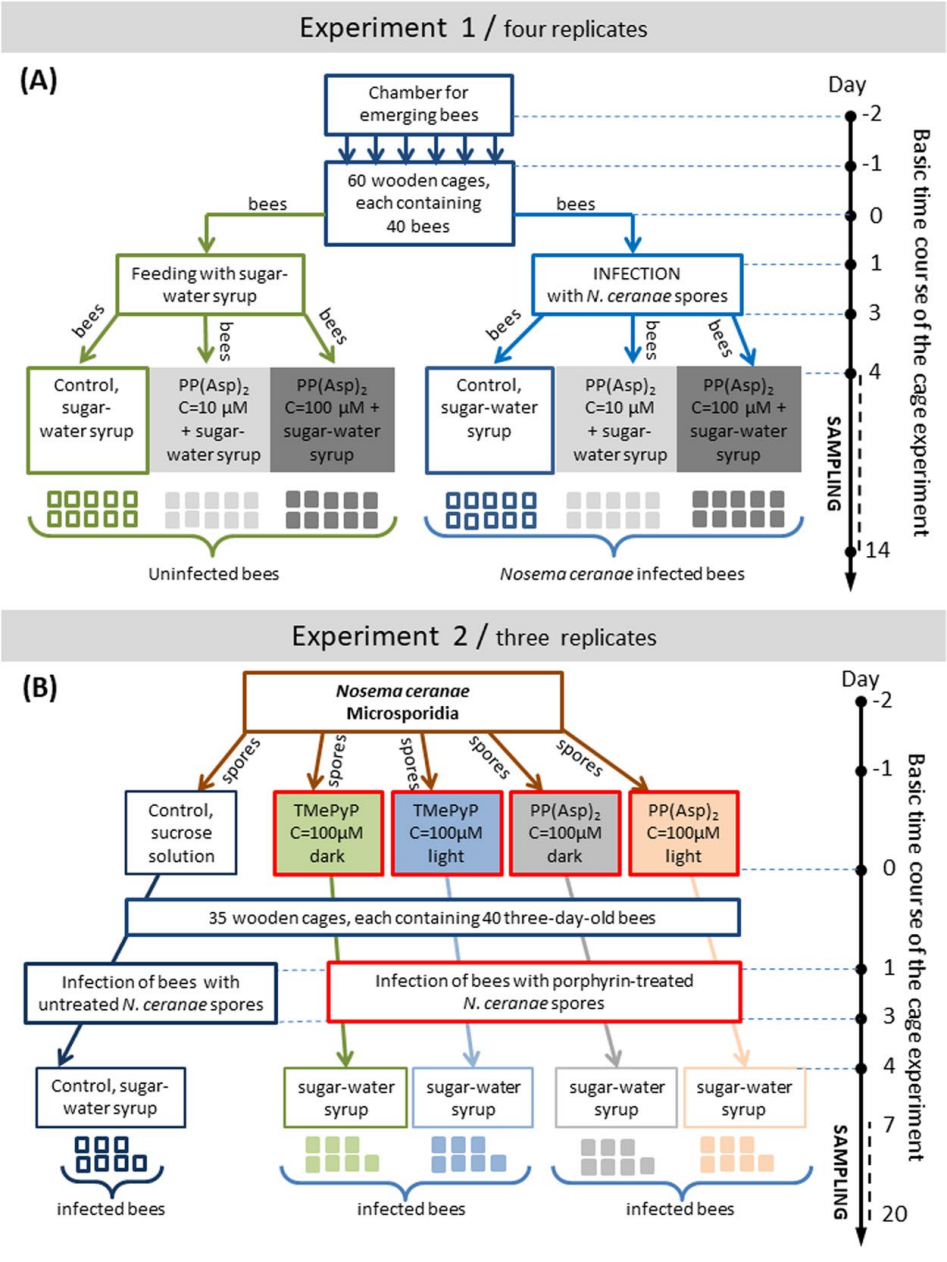
Two independent experiments analysing (Experiment 1) the effect of the porphyrin PP(Asp)_2_ on the number of *Nosema ceranae* spores in honeybees infected by microsporidia as well as on the survival of both healthy and *Nosema*-infected honeybees and (Experiment 2) the impact of *in vitro* incubation of the microsporidia in the porphyrin solutions (in the dark and under light irradiation) on the biological ability of the microsporidia to infect honeybees [level of infectivity].

To establish the role of light in the activation of PP(Asp)_2_ and TMePyP against microsporidia, two types of experiments were performed (Experiment 2). Microsporidia were treated (*in vitro*) with the porphyrin inhibitors^1^ in the dark or^2^ under light irradiation before being administered to the honeybees for infection (Fig. 2).

### Influence of porphyrin treatment on the course of nosemosis

**Figure 3:**
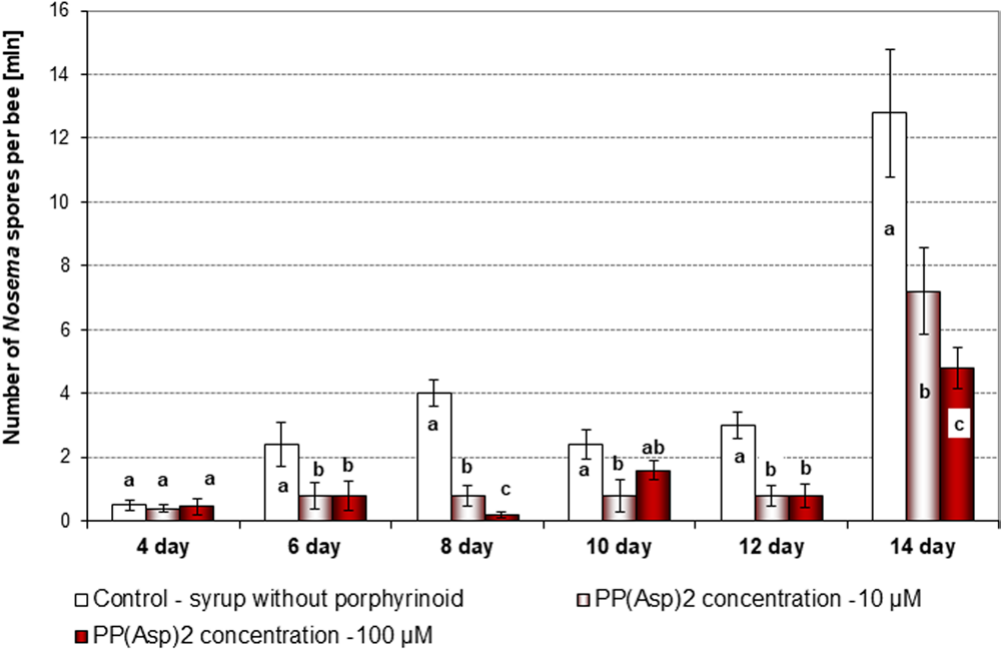
Effect of the porphyrinoid PP(Asp)_2_ on the number of spores in honeybees infected with microsporidia over 14 days of the experiment. These data represent Experiment 1, which was repeated four times. Statistically significant differences between the control group and the groups treated with PP(Asp)_2_ (10 µM and 100 µM) within a given day are indicated with lowercase letters: a, b, c; p ≤ 0.05 (ANOVA, Tukey test). Error bars represent standard errors of the data.

**Figure 4:**
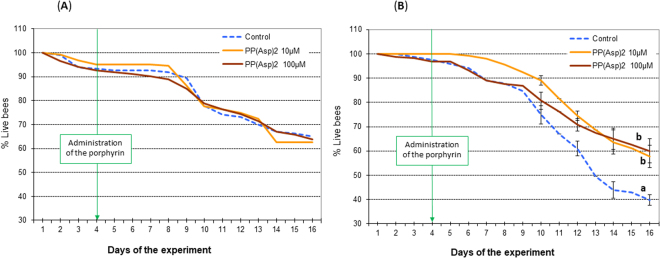
Effect of the porphyrinoid PP(Asp)_2_ on the mortality of the uninfected honeybees (**A**) and honeybees infected with microsporidia (**B**) in the cages over 12 days of the experiment. Lowercase letters (a, b) indicate significant differences between the control (bees fed pure sucrose syrup), PP(Asp)_2_ −10 µM and PP(Asp)_2_ −100 µM (bees fed with sucrose syrup supplemented with the porphyrin at concentrations of 10 and 100 µM, respectively) experimental groups (p ≤ 0.05) (ANOVA, Tukey test).

PP(Asp)_2_ was administered to the honeybees in a sucrose syrup supplement. The bees from all experimental groups consumed the syrup willingly and without apprehension (no preference for any of the syrup preparations was observed). The amount of syrup consumed by honeybees in all cages was on average 1.1 mL (s.e.m ±0.065) per 40 bees per day, with no considerable differences observed among groups. On day 1, the number of spores across all groups treated ranged from 0.13–0.145 × 10^6^ spores per bee. On day 4 of the experiment, the number of spores per bee increased to 0.4–0.5 × 10^6^; however, no significant changes (*F* = 1.420; p = 0.654) were observed in the number of spores across the groups studied (Fig. 3). All experimental data obtained for honeybees treated with PP(Asp)_2_ were compared to those obtained for the control honeybees, which were not treated with PP(Asp)_2_. From day 6, the number of *N. ceranae* spores among porphyrin-treated honeybees gradually decreased in comparison to the number of spores among control honeybees, which were given solely sucrose syrup (Fig. 3). On days 8 and 12 of the experiment, 5- and 4-fold reductions, respectively, were observed in the number of spores (*F* = 32.420, p = 0.021; and *F* = 19.212, p = 0.022, respectively). However, a rapid increase in the production of *Nosema* spores was observed from day 12 to day 14 in both the treated and control honeybees; nevertheless, the number of the spores was found to be 2.6-fold lower in the subjects treated with 100 µM porphyrin (*F* = 42.420; p = 0.01). Comparison of the number of deceased honeybees during the 12 days of the experiment after PP(Asp)_2_ administration indicated that there was no statistically significant increase in the mortality of uninfected honeybees after treatment with the porphyrin (Fig. 4A). Among *Nosema*-infected honeybees, we observed an increase in the lifespan of the bees treated with 100 μM porphyrin solution compared to the control bees (*F* = 12.024; p = 0.015) (Fig. 4B).

### Examination of the impact of *in vitro* porphyrin treatment on the ability of *Nosema* spores to infect honeybees

**Figure 5:**
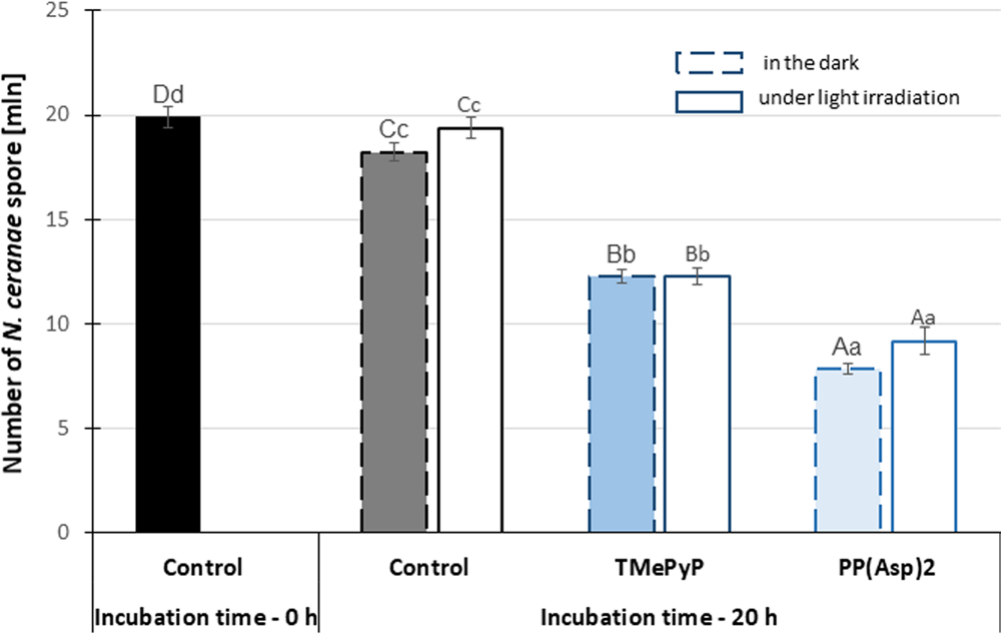
Reduction in the spore number after a 20-h (*in vitro*) incubation of the *Nosema ceranae* microsporidia with the porphyrins in the dark and under light irradiation. Uppercase letters (A, B, C, D) indicate that the differences among the control (0 h), control (20 h), TPyP (20 h), and PP(Asp)_2_ (20 h) groups are significant (p ≤ 0.01) (ANOVA, Tukey test). Lowercase letters (a, b, c, d) indicate that the differences among the control (0 h), control (20 h), TPyP (20 h), and PP(Asp)2 (20 h) groups are significant (p ≤ 0.05) (ANOVA, Tukey test). Error bars denote standard errors.

TMePyP and PP(Asp)_2_ were used to investigate the direct *in vitro* effects of porphyrins on *N. ceranae*. The spores were incubated for 20 h in the presence of either porphyrin, and both porphyrins were found to significantly reduce the number of microsporidia; TMePyP reduced the number of spores by 32% and PP(Asp)_2_ reduced the number of spores by 56% compared to the control (*F* = 124.24; p = 0.000) (Fig. 5). In the presence of light, the reduction in the number of *Nosema* spores increased slightly (up to 36.8%) after 20 h of incubation with TMePyP (*F* = 118.2; p = 0.000), while a reduction was not observed with PP(Asp)_2_.

**Figure 6:**
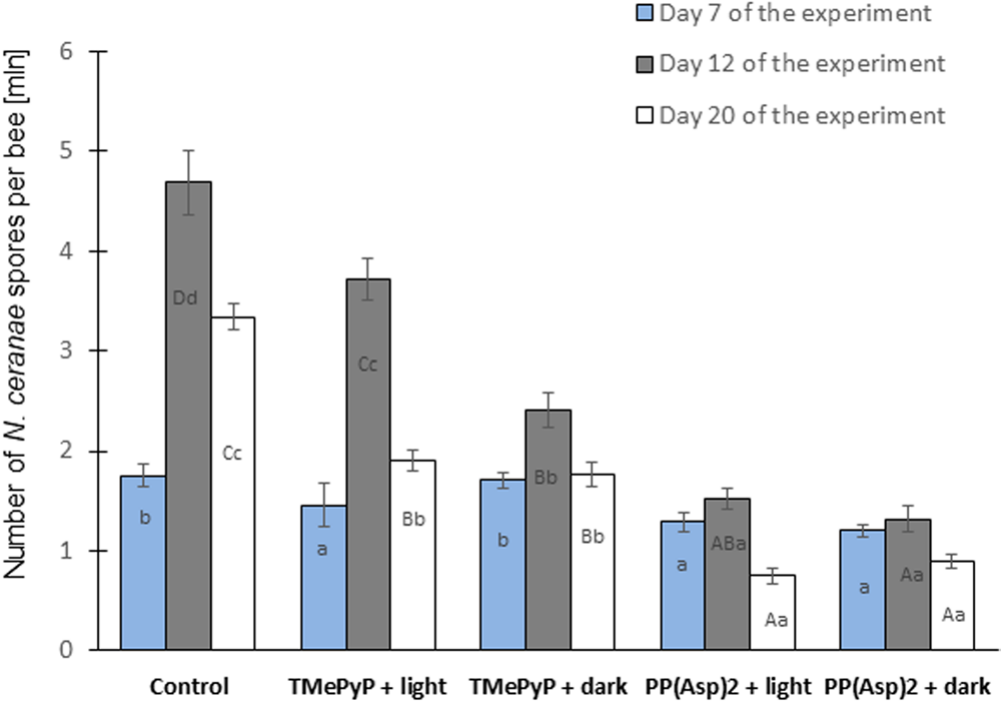
Impact of incubation of *Nosema ceranae* microsporidia in the porphyrin solutions on the number of spores in honeybees. Uppercase letters (A, B, C, D) indicate that the differences among the control, TMePyP + light, TMePyP + dark, PP(Asp)_2_ + light, and PP(Asp)_2_ + dark groups are significant (p ≤ 0.01) (ANOVA, Tukey test). Lowercase letters (a, b, c, d) indicate that the differences among the control, TMePyP + light, TMePyP + dark, PP(Asp)2 + light, and PP(Asp)2 + dark groups are significant (p ≤ 0.05) (ANOVA, Tukey test). Error bars denote standard errors.

**Figure 7:**
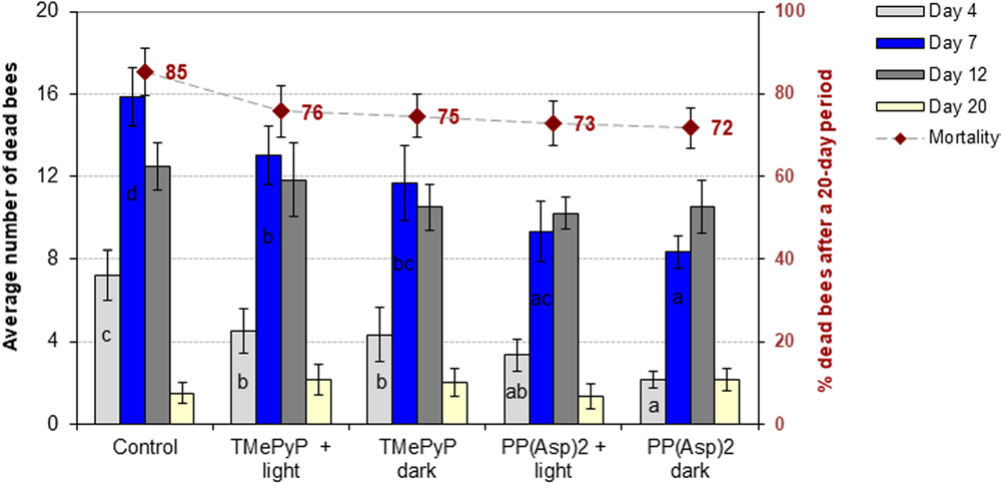
Mortality of honeybees after infection with *Nosema ceranae* microsporidia incubated in porphyrin solutions. Lowercase letters (a, b, c, d) indicate that the differences between the control groups and the TMePyP + light, TMePyP + dark, PP(Asp)_2_ + light, and PP(Asp)_2_ + dark study groups are significant (p ≤ 0.05) (ANOVA, Tukey test). Error bars represent standard errors of the data.

The infectivity of the pre-treated spores, i.e., the ability of these spores to infect honeybees, was examined. Honeybees exposed to preincubated spores treated with either TMePyP or PP(Asp)_2_ exhibited significantly decreased spore counts after days 12 and 20 compared to the honeybees in the control group, which were exposed to untreated microsporidia (*F* = 51.920, p = 0.000; *F* = 100.25, p = 0.000). In the absence of light, 1.9- and 3.7-fold reductions were observed in the number of spores on day 20 after treatment with TMePyP and PP(Asp)_2_, respectively, compared to the control (Fig. 6). Moreover, declining death rates of infected honeybees were also observed over the 20-day period. Lower mortality (72%) was observed in honeybees infected with PP(Asp)_2_-treated microsporidia compared to the control group of bees (85%) (Fig. 7). Statistically significant differences were observed on days 4 (*F* = 5.904; p = 0.0005) and 7 (*F* = 5.781; p = 0.0005) of the experiment. Exposure of spores treated with porphyrin PP(Asp)_2_ to light during the incubation period had no substantial effect on the ability of these spores to infect honeybees compared to the ability of the spores that were studied in the dark (Fig. 6).

### Morphology of the *Nosema ceranae* spores

**Figure 8:**
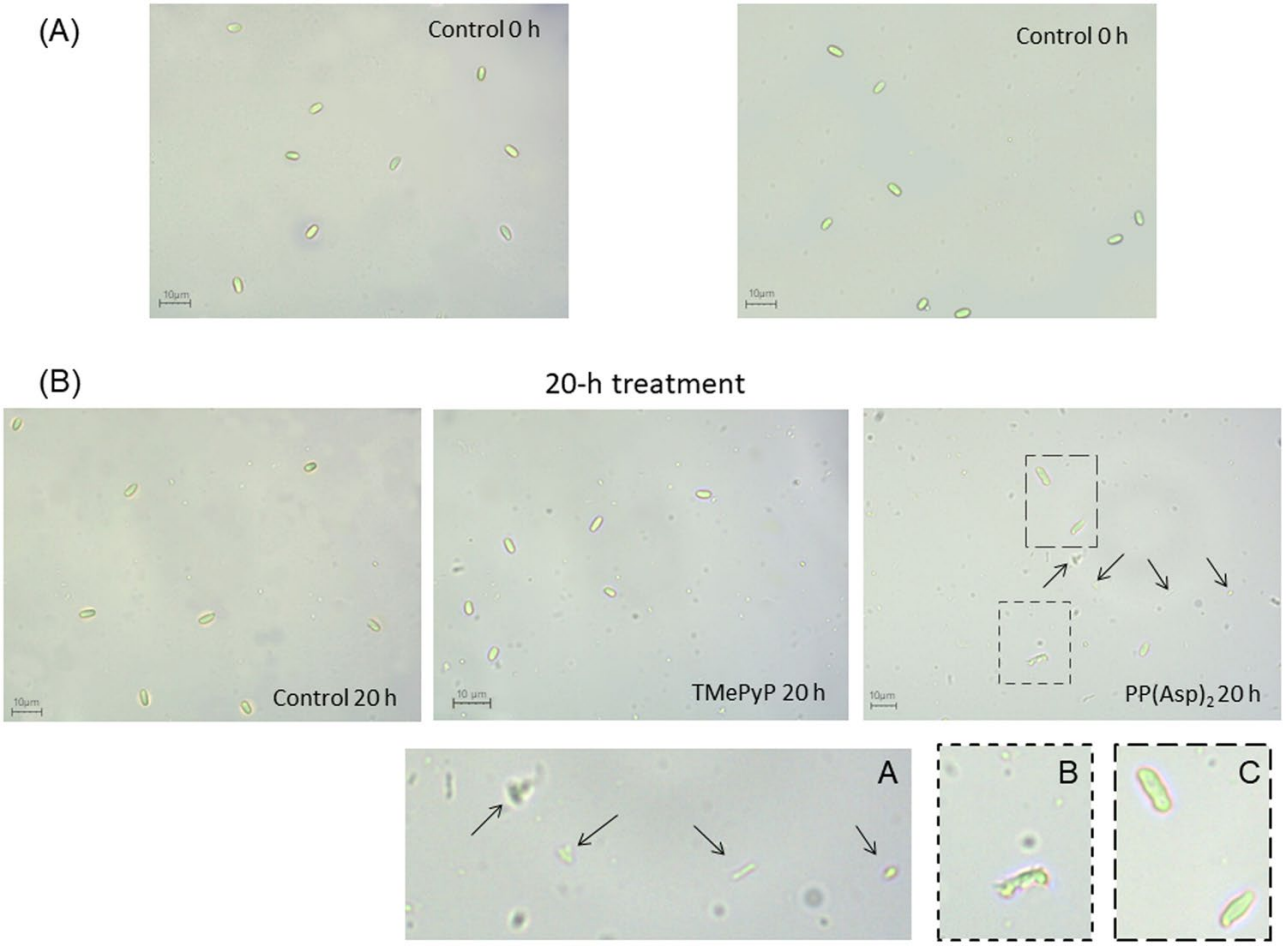
Light microscopic images of the microsporidia showing morphological differences between porphyrin-treated and untreated spore preparations. (**A**) At the beginning of incubation with porphyrins and (**B**) after 20 h of treatment. Morphological changes and cell debris are marked with dashed outlines and arrows (60× objective). Insets showing higher magnifications (dashed lines) are shown in panels (A,B) and (C). Spores with deformed walls are visible in panel (C).

Microscopic images of the microsporidia incubated (*in vitro*) in the porphyrin solutions or, as a control, in a pure 0.5% sucrose solution showed distinct differences in the morphologies of the microsporidia (Fig. 8). Greater morphological changes were observed in the images of spores treated with PP(Asp)_2_ than in the images of untreated spores. In addition, in contrast to the images of untreated or TMePyP-treated spores, numerous artefacts and cell debris were visible in the images of microsporidia treated with PP(Asp)_2_.

**Figure 9:**
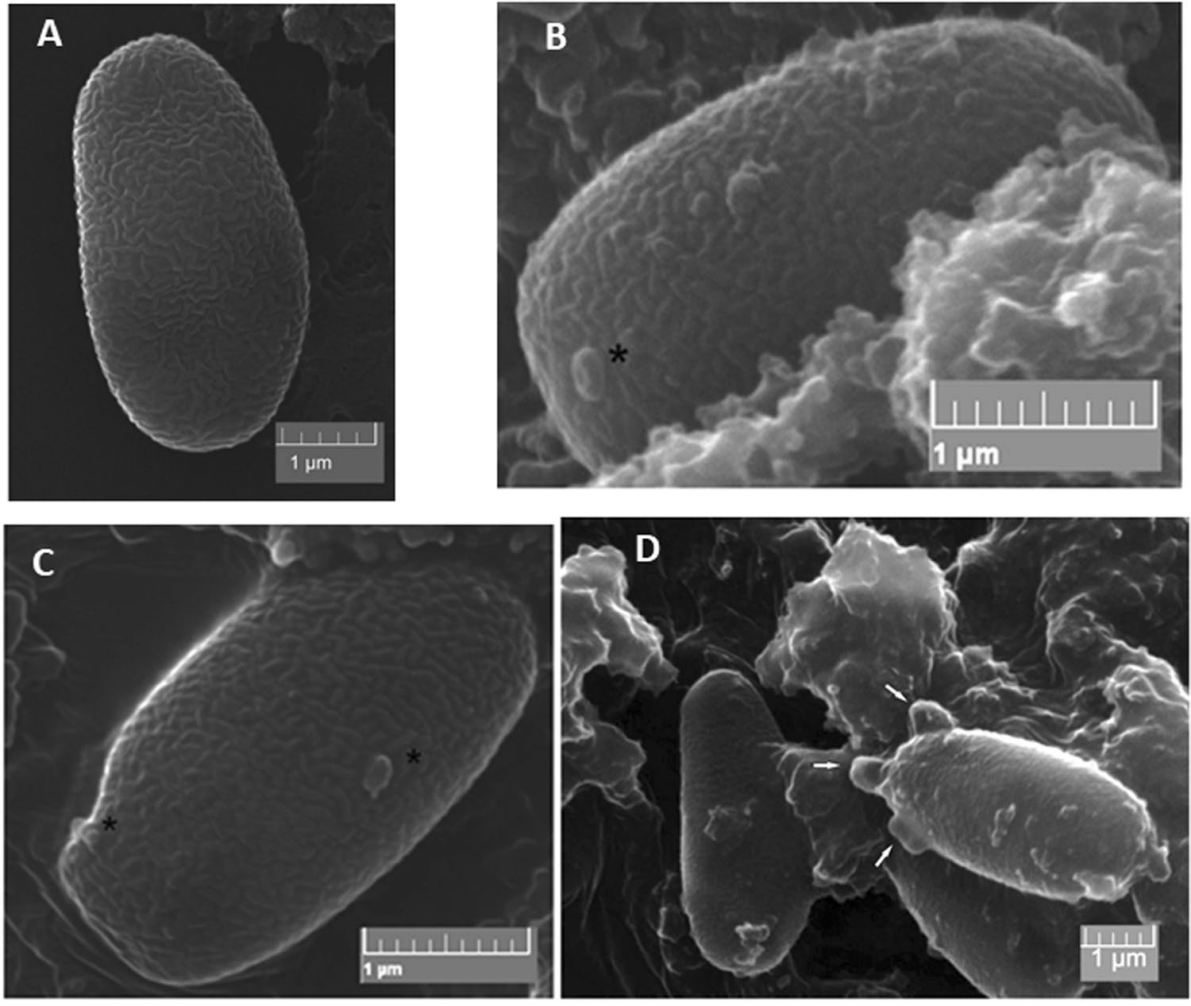
Changes in the spore exosporium observed by SEM. (**A)** Control; (**B**,**D**) spores treated with PP(Asp)_2_; (**C**) spores treated with TMePyP. Changes are marked with asterisks and arrows.

Scanning electron microscopy (SEM) allowed the study of the outer structure of the cell wall, especially of the exosporium layer^24^. Based on the SEM images, the morphology of the spores changed after exposure to porphyrinoid agents; the exosporium layers appeared deformed (Fig. 9). Some extreme exosporium deformities probably lead to the eruption of the microsporidial spores. In sharp contrast, in the control microsporidia, which were incubated in 0.5% sucrose solution lacking porphyrin, no deformities were observed.

## Discussion

The positive response of the honeybees treated with PP(Asp)_2_ in the sucrose syrup supplement indicated that the porphyrin is not toxic to honeybees. The amount of sucrose syrup consumed, with or without the porphyrin, was very similar (1.1 mL ±0.065) in all groups studied (including controls). Moreover, throughout the course of our cage experiments, the PP(Asp)_2_ dissolved in the sugar syrup was determined to have no adverse effects on honeybee lifespan (Fig. 4A). Importantly, the introduction of PP(Asp)_2_ into the diet of nosemosis-infected honeybees resulted in significant reduction (*F* = 19.212 and p = 0.022 on day 12) in the number of *Nosema* spores (Fig. 3). This phenomenon could be associated with the activity of PP(Asp)_2_ against microsporidia. Inactivation of the *Nosema* spores may prevent the development of these spores in infected honeybees. The effect of the different distributions of spore numbers consumed by individual bees in each cage may be excluded since at the beginning of the experiment (day 1), the number of spores across all groups studied were similar. On day 4, two days after infection, the spore numbers increased in a similar manner, but no substantial changes between the groups were recorded. Moreover, honeybees exchange food via a process called trophallaxis, which must have further compensated for the uneven infection of individual bees at the start of the experiment. A sharp increase in spore production in both treated and untreated bees was observed on day 14, presumably as a result of diminished immunity due to exposure of the insects to long-term stress (e.g., the stress caused by the absence of the queen bee).

Furthermore, the possibility of physicochemical changes in the properties of the porphyrin in the sucrose syrup may also affect the results. Nevertheless, a 2.7-fold reduction in spore count was observed after day 14 of the experiment in the group of honeybees treated with PP(Asp)_2_ (100 µM) compared to the spore count observed in the control group (*F* = 42.420; p = 0.001). Since the sucrose syrup was protected from light activation and the experiments were carried out in the dark, we conclude that PP(Asp)_2_ does not require light to exhibit activity against microsporidia.

In Experiment 2, we investigated the possibility of TMePyP and PP(Asp)_2_ acting directly on spores. This investigation was conducted by first treating the spores with either of the two porphyrins before exposing the spores to the honeybees. A positive correlation between the higher anti-microsporidial activity of the porphyrin, e.g., PP(Asp)_2_, characterized by increased cell wall penetration, and potential changes in the deformation of the microsporidial cell walls would indicate the direct action of porphyrinoids on microsporidia. Indeed, the results showed that both porphyrins, especially PP(Asp)_2_, reduced the infectious ability of the *Nosema* spores, causing significant inactivation of the microsporidia, preventing spore development (Fig. 6), and diminishing the mortality of infected honeybees (Fig. 7). Moreover, compared to the control, significant reduction in spore count was observed when the spores were first incubated *in vitro* with either of the porphyrins in the dark, with PP(Asp)_2_ being more effective than TMePyP (Fig. 5). However, in the presence of light, a slight decrease in PP(Asp)_2_ bioactivity was observed. This result may be associated with the negative effect of light irradiation on this compound, causing partial decomposition of the compound to inactive forms. Compared to the control (untreated spores), microscopic images of the microsporidial suspension sampled after 20 h of incubation with porphyrins showed distinctly higher numbers of deformed spores and various types of cellular debris, which were most likely a result of spore eruption (Fig. 8).

Furthermore, morphological changes in the exosporium layers of the PP(Asp)_2_-treated spores (Fig. 9) indicated the direct impact of the porphyrin on microsporidia. As the microsporidial spore wall consists of two layers: the exospore (or exosporium) and the endospore (or endosporium)^25–27^, it is reasonable to assume that this treatment would be beneficial against other pathogenic fungi from the phylum Microsporidia due to similarities in the structures of the exospores.

Notably, the porphyrins studied for their biocidal activity against microsporidia did not require light excitation. Porphyrins incubated with microsporidia in the dark and those incubated with microsporidia in the presence of light both clearly acted on the cell walls of the *Nosema* spores. Our findings regarding the bioactivity of metal-free porphyrins are dissimilar to previously reported data for other microorganisms. We assume that the differences between the structures of the external walls of bacteria and microsporidia lead to different mechanisms of the perturbance of these walls with porphyrins and ROS. In comparison to bacteria, microsporidia have anchoring disks, which are located at the thinnest ends of the cell walls^26,28^, allowing biomolecules to enter the cell. The porphyrins inside the spores may disrupt the microsporidia by inducing biochemical processes that disrupt the structural integrity of the spores, leading to deformities and ultimately to the destruction of the whole spore (Figs 8 and 9).

The anti-microsporidial activity of the nonmetalloporphyrins can be attributed to the perturbance of membrane and cell wall integrity by these porphyrins and is likely responsible for the inhibition of the synthesis of macromolecular compounds and for the death of microsporidia exposed to porphyrins^21,29^. The cytoplasmic membrane is considered the main cellular target of *Candida* photosensitization with porphyrins^30^. Similar findings have been reported for bacteria, in which the affinity of these compounds to external structures (e.g., cell walls and cytoplasmic membranes) is also thought to be both the main determinant of the efficiency of antimicrobial photosensitization^31^ and light-independent with respect to the *anti*-staphylococcal activity of XF-73^20^. Substantial damage to the exosporium is evident in the SEM images of the microsporidia treated with porphyrins (Fig. 8), which is consistent with our hypothesis. Damage to external microbial structures can result in the leakage of intracellular components or to the inactivation of membrane transport systems and enzymes^14^. Although the microorganism studied here is different from the microbial systems described above, it is likely that the targets of PP(Asp)_2_ might differ from those of the bioactive compounds that are currently used against microsporidia. In this context, the influence that other amino acid moieties on the porphyrin structure might have on penetration by the porphyrin of the two layers of the microsporidian spore wall and on the access of the porphyrin to intracellular targets should be comprehensively studied.

In addition to photodynamic inactivation, porphyrin compounds may also stimulate the production of ROS *in vivo*, which has been previously considered to be the source of metalloporphyrin-dependent antimicrobial activity against aerobically grown bacteria^18^. It is rather unlikely that such a mechanism operates in the environments of microsporidia since these microorganisms do not require oxygen to be metabolically active in their cell cycle (due to the absence of the mitochondria). The excitation of the porphyrins in the sugar syrup by photons should not be taken into consideration as the syrup was protected from light. However, even if some photons had acted on the porphyrin, ROS generated by excited porphyrins are short-lived in solution and would have probably been quenched before reaching the bee midgut. For example, singlet oxygen has a relatively long lifetime in organic solvents, such as chloroform and carbon tetrachloride (~230–700 µs), but in EtOH and water, the lifetime of singlet oxygen is much shorter, 15.3 and 3.5–5 µs, respectively^32,33^. Moreover, in viable, metabolically functional and H_2_O-containing cells, the lifespan of singlet oxygen is approximately 3 µs^34^, allowing the oxygen to travel only approximately 0.1 μm^35^. Due to the very short lifetime of singlet oxygen in condensed media and cellular environments, ROS (and excited porphyrin molecules) are most likely quenched to their electronic ground states before reaching the honeybee intestine. As a result, ROS cannot be considered to be factors that lead to the reduction of spore numbers.

However, another plausible mechanism, involving the inhibition of methionine aminopeptidase type-2 (which is responsible for the antifungal activity of fumagillin, an antibiotic used in microsporidiosis treatment) in microsporidia and inhibition of the life cycles of microsporidia by porphyrins should also be considered in future studies.

## Materials and Methods

### Chemicals

The protoporphyrin IX amide PP(Asp)_2_ was synthesized from protoporphyrin IX as described by Maximova *et al*.^36^ and fully characterized. ^1^H NMR (500 MHz, TFA-d_1_): δ = 11.11 (s, 2H, *meso*), 11.05–11.04 (m, 2H, *meso*), 8.33–8.26 (m, 2H, -CH=(vinyl)), 6.63 (dd, *J* = 11.5, 8.4 Hz, 1H, =CH_2_ (vinyl)), 6.43 (dd, *J* = 17.7, 5.3 Hz, 2 H, =CH_2_ (vinyl)), 4.77 (br s, 6 H, por-CH_2_-, CH-), 3.87 (s, 3 H, por-CH_3_), 3.84–3.83 (m, 6 H, por-CH_3_), 3.80 (s, 3 H, por-CH_3_), 3.32–3.26 (m, 4 H, -CH_2_-CONH-), 2.77–2.69 (m, 2H, CH_2_-), 2.59–2.46 (m, 2H, -CH_2_-) ppm. ^13^C NMR (125 MHz, TFA-d1): δ = 177.9, 177.8, 177.7, 176.9, 144.7, 144.5, 144.3, 144.2, 143.7, 143.6, 143.3, 143.2, 143.1, 142.2, 142.34, 142.31, 142.2, 142.1, 142.0, 141.9, 130.4, 130.3, 129.1, 129.0, 102.9, 102.1, 101.9, 100.6, 68.3, 50.8, 38.8, 38.7, 36.5, 24.24, 24.22, 14.7, 13.19, 13.18, 12.74, 12.74 ppm. HRMS-ESI: m/z [M + H]^+^ calcd for C_42_H_45_O_10_N_6_: 793, 3197; found: 793.3190. UV/Vis (10% aq. HCl): λ_max_ (ε) = 411 (2.45 × 10^5^), 557 (1.5 × 10^4^), 603 nm (5.00 × 10^3^). The purity of the porphyrin was ≥95%.

5, 10, 15, 20-Tetrakis(1-methylpyridinium-4-yl)porphyrin tetra(*p*-toluenesulfonate) (TMePyP), which was used in Experiment 2, was purchased from Sigma-Aldrich. The molecular structures of PP(Asp)_2_ and TMePyP are illustrated in Fig. 1.

The stock (2 mM) solution of the porphyrin in water was sterilized by filtration through a sterile filter (pore size, 0.22 μm) and stored in complete darkness until being added to the sucrose-water syrup (1:1 w/w), i.e., the sugar syrup used to feed the honeybees.

### Effect of the porphyrin PP(Asp)_2_ on the course of *Nosema ceranae* infection (Experiment 1)

During two beekeeping seasons, four independent cage experiments were conducted on summer honeybees as repetitions within Experiment 1 (two experiments were performed in 2014, and two in 2015). *Apis mellifera carnica* workers were gathered between June and July. During the summer, combs of broods originating from a single queen bee were taken on day 20 of their development, placed in an environmental chamber, and maintained at a constant temperature of 35 °C and a relative humidity of 60% to obtain 1-day-old healthy honeybees, which were tested for the presence of *N. ceranae* DNA as described in section ‘PCR-based assay to detect *Nosema* species’. These honeybees were maintained under laboratory conditions in complete darkness (25 °C; H = 65%) in 60 wooden cages, each occupied by 40 bees. Three days post-emergence, honeybees with *Nosema*-free intestines were randomly divided into two groups: 1) uninfected honeybees and 2) honeybees to be infected with *N. ceranae* spore-containing solution (5 × 10^6^ spores/mL)^37^. The honeybees were infected by feeding them sucrose-water syrup containing *N. ceranae* spores; the infection procedure was performed over two days, and the 1^st^ day of infection was labelled “day 1” of the experiment. The average quantity of spores administered to each bee was estimated from the total volume of spore-containing syrup consumed by all bees in a cage. A PCR assay to detect *Nosema* species (section ‘PCR-based assay to detect *Nosema* species’) was performed to confirm the presence of *N*. *ceranae* DNA in the infected honeybees. On day 4 of the experiment, both infected and uninfected honeybees were given a fresh supply of sucrose-water syrup (1:1 w/w) supplemented with PP(Asp)_2_ (at concentrations of 10 and 100 μM). The control subjects (in the infected and uninfected groups) were given only pure sucrose syrup, which was administered to the honeybees via syringes containing 5 mL of the syrup. Thus, six experimental groups were obtained with 10 cages each (Fig. 2). To verify the level of infection, i.e., the number of *N. ceranae* spores, every 2^nd^ day (days 4, 6, 8, 10, 12 and 14 of the experiment), two independent samples were prepared from each of the six experimental groups. Ten whole honeybees (one specimen from each cage) were pooled, and the pooled sample was homogenized in 10 mL of sterile, distilled water. The number of *N. ceranae* spores was then counted according to the method described by Hornitzky^38^ and Fries *et al*.^39^. Estimation of the number of *N. ceranae* spores per honeybee was accomplished using a haemocytometer and an Olympus BX61 light microscope. Additionally, the number of deceased honeybees was recorded during the experiments.

### Impact of pre-treatment of *Nosema ceranae* spores with the PP(Asp)_2_ and TMePyP porphyrins on the ability of the microsporidia to infect honeybees (Experiment 2)

During one beekeeping season (May-August 2016), three independent cage experiments were conducted on summer honeybees for Experiment 2. Prior to infection with porphyrin-treated or untreated spores, the honeybees were handled and maintained under the conditions described in section ‘Effect of the porphyrin PP(Asp)_2_ on the course of *Nosema ceranae* infection (Experiment 1)’. The intestines from 30 *N*. *ceranae*-infected honeybees were isolated to obtain a spore solution. The presence of the *N*. *ceranae* DNA in the solution was demonstrated by molecular detection of the specific 16S rDNA using a PCR-based method (section ‘PCR-based assay to detect *Nosema* species’). After isolation, the intestines were homogenized in 0.1 M McIlvaine buffer and filtered through a sterile mesh. Then, the fresh suspension of spores was washed twice with sterile phosphate-buffered saline (PBS), and the concentration of the spores was adjusted to 2 × 10^7^ mL^−1^ in 0.5% sucrose solution containing 0.005% Tween 80. Subsequently, the spore suspension was divided into three parts^1^: the spores were suspended in 25 mL of a 0.5% sucrose solution containing PP(Asp)_2_ (100 μM)^2^; the spores were suspended in a sucrose solution containing TMePyP (100 μM); and^3^ the spores were left untreated in the same volume of solution as the control. These three spore suspensions were incubated on a rotary shaker (160 rpm) for 20 h at 25 °C (replicating the temperature of the honeybee colony) under two conditions: under visible light and in the dark to avoid phototoxic effects, which could contribute to the anti-sporidial activity of the porphyrins.

In photoactivation experiments, the glass vessels containing microsporidia were evenly irradiated by four fluorescent visible lamps (Osram Lumilux, Cool White) for 20 h with a controlled intensity of light (irradiance of 140 μmol/m^2^ s). After the specified time, the spores were centrifuged for 15 min at 4,000 rpm and washed extensively using a sterile, aqueous 0.9% sodium chloride solution and then a 0.5% aqueous sucrose solution. The procedure was repeated four times to remove any porphyrin residues. All spore pre-treatments were carried out in parallel with the controls that were under exactly the same conditions but used a fresh spore suspension devoid of porphyrin. The spore solutions obtained were divided into three portions: (1) the first portion was mixed with sucrose syrup and used to infect honeybees; (2) the second portion was used to estimate the number of spores and image the spores by light microscopy (Nikon ECLIPSE E200); and (3) the third potion was used for SEM analysis (section ‘Sample preparation for scanning electron microscopy (SEM)’). Emerging honeybees with *Nosema*-free intestines (see section ‘Effect of the porphyrin PP(Asp)_2_ on the course of *Nosema ceranae* infection (Experiment 1)’) were divided randomly into five groups with 7 cages each. The differences in infectivity of pre-treated and untreated spores were determined by measuring the number of the spores developed in the honeybees. In two groups, the honeybees were infected with the spores that were pre-treated with porphyrins in the dark. In two other groups, the specimens were infected with porphyrin-treated spores preincubated under light irradiation. Honeybees in the control group were infected with *N. ceranae* spores that were not treated with porphyrin. Prior to infection, the spore suspensions obtained were adjusted to have equal concentrations, and the method of honeybee infection was the same as that used in Experiment 1. On days 7, 12 and 20 of the experiment, the level of infectivity of the pre-treated and untreated spores was estimated by counting the microsporidial spores that developed in the honeybees as described in section ‘Effect of the porphyrin PP(Asp)_2_ on the course of *Nosema ceranae* infection (Experiment 1)’. In Experiment 2, ten whole honeybees (one specimen from each of the seven cages and three additional bees randomly selected from the study population), were homogenized in 10 mL of sterile, distilled water, and the number of *N. ceranae* spores was counted according to the method described by Hornitzky^38^ and Fries *et al*.^39^.

In all the experiments performed to determine the light-independent activity of the porphyrins, the solutions were protected from light by maintaining the cages and performing *in vitro* experiments in the dark. Alternatively, when needed, dimmed lighting or lamps emitting red light were used.

### Sample preparation for scanning electron microscopy (SEM)

Each microsporidial spore sample was fixed in 5% glutaraldehyde (v/v) in 0.1 M phosphate buffer (pH 7.3) for 24 h. Then, the samples were washed with the phosphate buffer prior to fixation with 1% osmium tetroxide in 0.1 M phosphate buffer for 24 h, which was followed by washing with the same buffer. SEM samples were dehydrated by immersion for 15 min each in fresh solutions of 30%, 50%, 75%, 90%, and 100% acetone and critical point dried. The dried samples were mounted on specimen stubs using double-sided adhesive tape and coated with gold. Coated samples were viewed under a VEGA LMU scanning microscope at 30 KV, measured, and photographed.

### PCR-based assay to detect *Nosema* species

Spore suspensions or honeybee homogenates (100 μL) were added to lysis buffer (180 μL) and proteinase K (20 μL), and the DNA was isolated by the DNeasy Blood and Tissue Kit (Qiagen) according to the manufacturer’s instructions. Each isolate was used as a template for the detection of *N*. *apis*- and *N*. *ceranae*-specific 16S rDNA by PCR with *Nosema*-specific primers: 321-APIS for *N*. *apis* and 218-MITOC for *N*. *ceranae*^40^. Each spore suspension used to infect the honeybees in Experiments 1 and 2 was tested in triplicate for the presence of *N*. *apis*- and *N*. *ceranae*-specific 16S rDNA by PCR.

### Statistical analysis

Statistical analysis was performed using SAS software version 9.5 (Statistical Analysis System Institute, Cary, NC). Comparisons among the control and porphyrin groups [PP(Asp)_2_; TMePyP] on each day (Figs 3, 4, 6, 7) or incubation time (Fig. 5) of the experiments were performed using one-way ANOVA and the Tukey test. The data are reported as the mean values of all replicates. Differences of p ≤ 0.05 were considered significant.

## Conclusions

1. Porphyrins, in this case amide derivatives of PPIX, are promising candidates for combating microsporidiosis, especially nosemosis, in honeybees as they cause substantial reduction in spore numbers.
2. Notably, we have clearly shown that porphyrins used against microsporidia do not require exposure to light.
3. It was determined that less infectious *Nosema* spores may be obtained by preincubating the spores with the porphyrin prior to honeybee exposure.
4. Although our investigations into the mode of action of these porphyrins revealed deterioration of the cell wall, further in-depth studies must be and are currently being conducted.
